# Evaluating Cellular Polyfunctionality with a Novel Polyfunctionality Index

**DOI:** 10.1371/journal.pone.0042403

**Published:** 2012-07-30

**Authors:** Martin Larsen, Delphine Sauce, Laurent Arnaud, Solène Fastenackels, Victor Appay, Guy Gorochov

**Affiliations:** 1 Institut National de la Santé et de la Recherche Médicale (Inserm) UMR-S 945, Paris, France; 2 Université Pierre et Marie Curie - Univ Paris 06, Paris, France; 3 Service de médecine interne, AP-HP, Hôpital Pitié-Salpêtrière, Paris, France; 4 Laboratoire AP-HP d'Immunologie Cellulaire et Tissulaire, Paris, France; National Institute of Infectious Diseases, Japan

## Abstract

Functional evaluation of naturally occurring or vaccination-induced T cell responses in mice, men and monkeys has in recent years advanced from single-parameter (e.g. IFN-γ-secretion) to much more complex multidimensional measurements. Co-secretion of multiple functional molecules (such as cytokines and chemokines) at the single-cell level is now measurable due primarily to major advances in multiparametric flow cytometry. The very extensive and complex datasets generated by this technology raise the demand for proper analytical tools that enable the analysis of combinatorial functional properties of T cells, hence polyfunctionality. Presently, multidimensional functional measures are analysed either by evaluating all combinations of parameters individually or by summing frequencies of combinations that include the same number of simultaneous functions. Often these evaluations are visualized as pie charts. Whereas pie charts effectively represent and compare average polyfunctionality profiles of particular T cell subsets or patient groups, they do not document the degree or variation of polyfunctionality within a group nor does it allow more sophisticated statistical analysis. Here we propose a novel polyfunctionality index that numerically evaluates the degree and variation of polyfuntionality, and enable comparative and correlative parametric and non-parametric statistical tests. Moreover, it allows the usage of more advanced statistical approaches, such as cluster analysis. We believe that the polyfunctionality index will render polyfunctionality an appropriate end-point measure in future studies of T cell responsiveness.

## Introduction

Evaluating qualitative and quantitative phenotypic properties of T cell responses to pathogens [1], [2], [3], [4], [5], [6], [7], [8], [9], [10], [11], allergens [12], [13], [14] and mitogens [15] have had a tremendous impact on a large number of clinical studies, such as vaccination protocols. [16], [17], [18]

Early studies made use of ELISA techniques to measure cytokine secretion from pools of cells. Evaluating the frequency of functional cells was later made possible by the introduction of ELISPOT technology [19] and intra-cellular staining approaches. These techniques were first used to evaluate a single cytokine, such as interferon-γ (IFN-γ) at the single-cell level. Technical improvements later made it possible to observe the secretion of multiple cytokines at the single-cell level. [20], [21] Presently, state of the art multiparametric flow cytometry analysis enables the simultaneous detection of a large number (routinely n>10) of parameters at the single-cell level. The technique enables phenotypic and functional evaluation of biologically pertinent common (e.g. CD4, CD8, B and NK) and rare (e.g. pathogen specific) cell subsets defined by several parameters, such as surface markers and T- and B-cell receptor specificities. [21], [22] The focus on acquisition and analysis of increasingly multiparametric datasets continues to drive technological advances, such as mass cytometry Time-of-Flight [23] and chip based single-cell secretomics. [24]

Flow cytometry has largely facilitated the acquisition of enormous datasets. However, data analysis and presentation has become increasingly laborious and time consuming. Due to this limitation many studies long reported on individual functional parameters rather than combinations of functional parameters. The fastidious data analysis was recently substantially simplified by the development of pivotal analysis software, such as Spice, [25] which facilitates the visualisation of complex multiparameter datasets.

Recent works have highlighted the importance of multiparametric functional T cell assessment in studies concerning vaccine efficacy [26] and immunological control of cancer outgrowth, [24], [27], [28], [29] autoimmunity, [30], [31] and viral replication (e.g. Human immunodeficiency Virus (HIV) [3], [32], [33], [34], [35], Simian Immunodeficiency Virus (SIV) [36] and herpesviruses [37]). Multiparametric functional assessment of T cells can be presented without loss of information as the frequency of each combinatorial functional T cell subsets (2^n^ combinations), but commonly the 2^n^ dimensions are reduced to n dimensions to unify the data in a polyfunctionality profile frequently depicted as a pie chart, where the n^th^ pie slice represents the frequency of cells performing n simultaneous functions. However, at present no tool exists that will reduce the n-dimensional polyfunctionality profiles to a one-dimensional index value. Although such a reductionist approach inevitably results in a loss of information, it would largely benefit from the numerous analytical tools exclusively compatible with one-dimensional values. Indeed, a polyfunctionality index would allow an examination of the degree and variation of polyfunctionality within a particular dataset, and facilitates parametric and non-parametric statistical comparisons, correlations as well as hierarchical cluster analysis of polyfunctionality and other clinical or biological pertinent parameters.

In this study we describe a novel polyfunctionality index that enumerates cellular polyfunctionality as a one-dimensional value. The index applies to an unlimited number of functional parameters and can be employed in classical statistical analysis. To illustrate the strength of the novel polyfunctionality index, we have applied it to a set of multiparametric flow cytometry data, and show that it facilitates the analysis of T cell polyfunctionality. More precisely, we demonstrate how the index easily applies to comparative and correlative studies and enables more sophisticated biostatistical analysis, such as hierarchical cluster analysis. Additionally, the relative impact of polyfunctionality in particular clinical and biological contexts is determined numerically by automated iterative calculations. The polyfunctionality index presented in this study will be valuable for an increasingly large number of biological and clinical studies, where polyfunctionality represents a crucial end-point measurement.

## Results

### Design of a polyfunctionality index

T cell polyfunctionality has been analyzed in a large number of studies both by us [3], [4], [11], [15], [38], [39] and others. [7], [9], [40], [41], [42] These studies all use representative pie charts to illustrate the polyfunctionality of T cells, where the n^th^ pie slice represents the frequency of T cells with n simultaneous functions. The polyfunctionality profile is then compared between T cell subsets or clinically distinct donor groups by pie chart comparison statistics. [25] The statistics employed sequentially compare pie slices to globally evaluate if a pie chart differs from another. However, the statistical evaluation does not directly reflect differences in the degree of polyfunctionality, but rather differences in the profile of polyfunctionality. Presently, the degree of polyfunctionality can only be evaluated by a human interpretation, subjectively applying weights of importance to individual pie slices. Due to the impracticality of generating individual pie charts for each data point, pie charts commonly represent the average polyfunctional profile of a range of data points. Therefore pie charts are incompatible with methods of evaluating the variation of individual datasets and the application of more sophisticated statistical analysis, such as correlative measures and hierarchical cluster analysis. Our goal was therefore to develop a one-dimensional polyfunctionality index that would reflect the degree of polyfunctionality of a cellular subset and be compatible with a large spectre of statistical and bioinformatical techniques.

**Figure 1:**
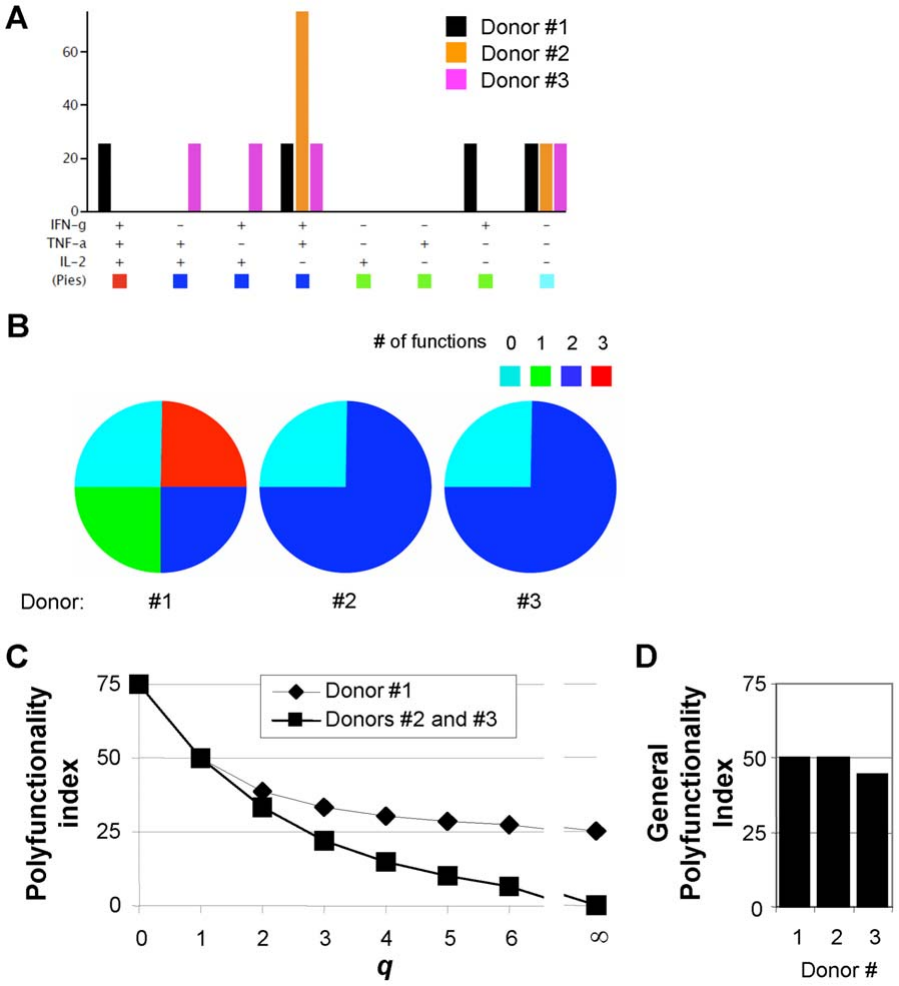
Theoretical example of polyfunctionality analysis of 3 fictive donors. **A,** Bar diagram indicating the frequency of T cells secreting each of the 8 (2^3^) combinations of IFN-γ, TNF-α and IL-2 for three theoretical functional T cell profiles. **B,** Pie chart representations of the functional T cell profiles. Pies represent the capacity of T cells to secrete none (0) or any (1, 2 or 3) of the three cytokines IFN-γ, TNF-α and IL-2. E.g. the red pie slice indicates the proportion of cells producing three cytokines (IFN-γ, TNF-α and IL-2). **C,** The polyfunctionality index depicted for each donor as a function of the parameter *q* defined in equation (1). **D,** The general polyfunctionality index (Appendix S1) applied to the polyfunctionality profiles of the three donors for *q* = 1 and φ_IFN-γ_ = 0.5 (φ_TNF-α_ = φ_IL-2_ = 0).

As an example, imagine monitoring the capacity of CD4^+^ T cells to secrete IFN-γ, TNF-α and IL-2 upon T cell stimulation (Figure 1A). One donor (Donor #1) presents a functional phenotype where 25% of CD4^+^ T cells secrete all three cytokines, 25% secrete 2 of the three cytokines, 25% secrete 1 of the three cytokines and the last 25% secrete none of the three cytokines. Another donor (Donor 2# or #3) presents a functional phenotype where no T cells secrete all three cytokines, 75% secretes 2 of the three cytokines, 0% secretes 1 of the three cytokines and 25% secretes no cytokines (Figure 1B). The difference between Donor #2 and #3 is that the 75% double functional T cells consist of IFN-γ^+^TNF-α^+^IL-2^−^ T cells or equal frequencies of IFN-γ^−^TNF-α^+^IL-2^+^, IFN-γ^+^TNF-α^−^IL-2^+^ and IFN-γ^+^TNF-α^+^IL-2^−^ T cells, respectively (Figure 1A). A pie chart of donor #1 would consist of 4 pie slices of equal size, whereas donor #2 and #3 would have a pie chart consisting of 2 pie slices of which one represents 75% of the pie (Figure 1B). Pie chart statistics would clearly distinguish the polyfunctionality profiles of donor #1 and #2. However, objectively evaluating which donor has the more polyfunctional CD4^+^ T cells is difficult. First question is how to weigh non-, mono- and poly-functional subsets. A conservative approach would be to weight them in a linearly increasing manner. However, e.g. if vaccine studies would show that only triple functional CD4^+^ T cells are protective, one could consider grading the weights in a non-linear (e.g. exponential) manner, which would more strongly favour triple functional CD4^+^ T cells over less polyfunctional CD4^+^ T cells. The second question is whether some measured functionalities are more important than others. E.g. a vaccination protocol could consider the secretion of IFN-γ more important for protection than secretion of IL-2, in which case Donor #2 should be considered superior compared to Donor #3 (Figure 1A). Therefore defining a general algorithm is challenging. We have chosen the algorithm (1)


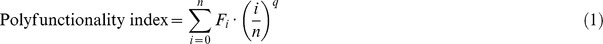



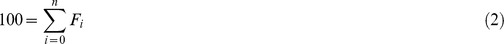


and


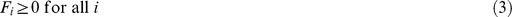


where *n*>0 is the number of functions studied, *F_i_* is the frequency (%) of cells performing *i* functions and *q*≥0 is the parameter that modulates the differential weight assignment of each F_i_. The algorithm requires that the sum of all F*_i_* equals 100 (2) and that all F*_i_* are absolute values (3).

Testing the extremities of this equation it becomes evident that the polyfunctionality index is restrained to values between 0 and 100 (visualized for *n* = 2 in Figure S1A). A polyfunctionality index of 0 occurs when all cells are non-functional (*F_0_* = 100), and a polyfunctionality index of 100 occurs when all cells have the maximum number of functions measured (*F_n_* = 100). As expected from such an algorithm, the polyfunctionality index for *n* = 1 becomes identical to the frequency of cells secreting the one cytokine measured. Contrarily, for large *n*'s the polyfunctionality index is primarily defined by the cells performing multiple functions, as contributions from less polyfunctional T cells (small *i*) becomes insignificant. This is reasonable also in a biologic setting, as the chance of a cell to perform at least one function increases with the number of functions measured. It has been suggested that future analytical tools should enable the possibility of extensively favouring highly polyfunctional T cells over less polyfunctional T cells. [25] Indeed, the polyfunctionality index (1) presented here allows us to favour more polyfunctional T cells in a linearly (*q* = 1) or exponentially (*q*>1) increasing manner, according to the biology of the functionalities measured.

Taking the example from above where three cytokines (*n* = 3) are measured and setting *q* to 1 (the most conservative value) all three donors described have a polyfunctionality index of 50 (Donor #1: PI = 0/3×25%+1/3×25%+2/3×25%+3/3×25% = 50 and donors #2 and #3: PI = 0/3×25%+1/3×0%+2/3×75%+3/3×0% = 50). Thus, the algorithm considers that T cells where 75% of the cells secrete 2 cytokines are as polyfunctional as T cells where 1, 2 and 3 simultaneous cytokines are secreted by each 25% of the T cells. Thus, different polyfunctionality profiles may describe T cells with identical degree of polyfunctionality. This is logic both mathematically and immunologically. Mathematically because reducing n-dimensional polyfunctionality profiles to a one-dimensional degree of polyfunctionality inevitably leads to a loss of information. And immunologically because T cells with a large frequency of cells performing n-1 functions may be as polyfunctional as T cells with a smaller frequency of cells performing n functions as illustrated with donor #1 and #2 above. We previously reported that the polyfunctionality of T cells is gradually modulated by antigen sensitivity and antigen load. [3] The extreme theoretical examples shown in Figure 1 are therefore unlikely to occur in reality, particularly when measuring functionality of T cells. It is nonetheless important to consider these extreme cases to fully outline the conceptual difference between profiling and enumerating polyfunctionality. We have therefore calculated the polyfunctionality index for a fictive panel covering a large spectra of polyfunctionality profiles (n = 2, Figure S1B). The example illustrates the association between multiple different polyfunctionality profiles and one polyfunctionality index value.

Increasing *q* (*q* = 2) increases the weight of the cells that are more polyfunctional. Thus, in this case donor #1 has a polyfunctionality index of 39, whereas donors #2 and #3 have a polyfunctionality index of 33 (Figure 1C). We also illustrate this effect on the broad range of polyfunctionality profiles discussed above (Figure S1B). Of note, if *q* approaches zero the equation will evaluate any functional T cell irrespective of its polyfunctionality (Figure 1C). Donor #1, #2 and #3 would all have a polyfunctionality index of 75, corresponding to the sum of functional T cells. Conversely, increasing *q* augments the weight of more polyfunctional T cells. Indeed for infinite large *q* the equation evaluates polyfunctionality solely as the frequency of cells performing all measured functions (F_n_) (Figure 1C). Presently, no proper biological reason exists to defend an exponentially increased weight of highly polyfunctional T cells. Therefore, throughout this study we use the most conservative *q* (*q* = 1), where the weight of the individual polyfunctional subsets (*F_i_*) increases linearly with *i*.

Whereas the algorithm (1) brings a solution to the first question regarding the attribution of weights to individual pie slices for the calculation of the degree of polyfunctionality, it does not bring a method to discriminate the importance of one function from another (question 2). Indeed, the polyfunctionality index weighs all functionality measures equally. One can therefore not favour IFN-γ-secreting cells over IL-2-secreting cells, or in other words one can not distinguish between donors #2 and #3. To address this issue we have developed a “general polyfunctionality index”, for which the polyfunctionality index (1) above is a special case where all measured functions are considered equally important. The “general polyfunctionality index” allows the attribution of individual weights to each measured function, and thus would enable the discrimination of donor #2 and #3 (Figure 1D and Appendix S1). In the examples presented here there are no biological reasons for considering one function more important than the others. We therefore only present data using the polyfunctionality index (1).

In the following sections we will apply the polyfunctionality index to multiparametric flow cytometry profiles. These profiles are obtained from two of our previously published studies, [11], [15] as well as unpublished data on HIV specific functional T cell responses. These data efficiently illustrate the potentials of the polyfunctionality index.

### Application of the polyfunctionality index on IL-17A- and/or IL-22-secreting CD4^+^ T cells

**Figure 2:**
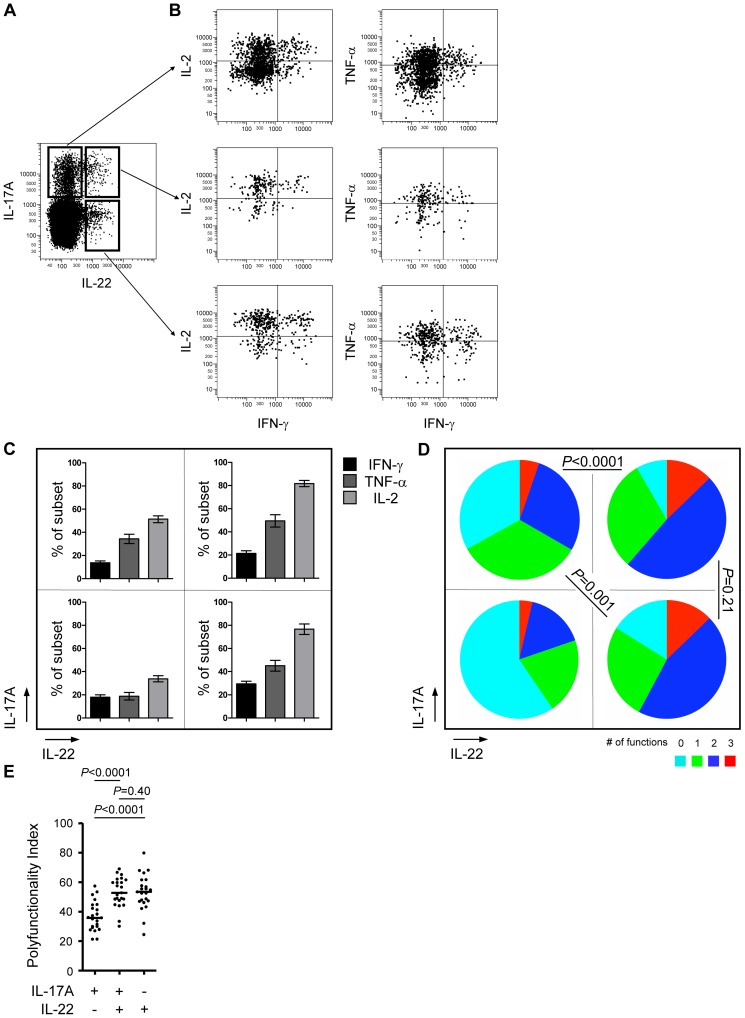
Characterization of IL-17A- and/or IL-22-secreting CD4^+^ T cells in healthy controls. **A,** Representative cytofluorometric analysis of IL-17A and IL-22 intra-cellular expression by peripheral CD4^+^ T cells following PMA/ionomycin-stimulation. **B,** Gating on IL-17A^+^IL-22^−^ (top panel), IL-17A^+^IL-22^+^ (middle panel) and IL-17A^−^IL-22^+^ (bottom panel) CD4^+^ T cells we then determined their co-secretion of 3 additional cytokines, IFN-γ (black bars), TNF-α (dark gray bars) and IL-2 (light gray bars). **C,** Bar diagrams depicting individual cytokine responses and **D,** Pie charts illustrating the polyfunctional profiles of the 4 combinatorial subsets of IL-17A/IL-22-secreting CD4^+^ T cells from 25 healthy controls. **E,** Corresponding polyfunctionality index values of IL-17A- and/or IL-22-secreting T cells. Horizontal lines indicate median frequencies, and *P*-values were calculated using the pie statistic tool integrated in the Spice software and the Wilcoxon signed rank test.

We previously studied the polyfunctional profile of proinflammatory CD4^+^ T cells secreting IL-17A and/or IL-22. [15] Here we extended these studies to 25 healthy individuals (Figure 2A). Polyfunctionality was defined as the capacity to secrete IFN-γ, TNF-α and/or IL-2 (Figure 2B). We observed that IL-22^+^ CD4^+^ T cells co-secrete TNF-α and IL-2 in larger proportions than IL-17A^+^IL-22^−^ CD4^+^ T cells, irrespective of their IL-17A status (Figure 2C), thus demonstrating that co-secretion of the latter cytokines is associated with IL-22- rather than with IL-17A-secretion. This resulted in significantly different polyfunctionality profiles of individual subsets according to pie chart statistics (Figure 2D). To properly assess the degree of polyfunctionality associated with IL-22-secreting versus IL-17A-secreting CD4^+^ T cells, we applied the polyfunctionality index to the three IL-17A/IL-22 CD4^+^ T cell subsets. Of note, IL-22-secreting CD4^+^ T cells irrespective of IL-17A-secretion are significantly more polyfunctional than IL-17A^+^IL-22^−^ CD4^+^ T cells using the non-parametric Wilcoxon signed rank test (*P*<0.0001, Figure 2E). Moreover, the variability of polyfunctionality within each cellular subset was easily scrutinized by analyzing distribution statistics, such as their respective range and confidence intervals. The 95% confidence interval of polyfunctionality for each of the three functional CD4^+^ T cell subsets were determined as [30.2–39.7] for IL-17A^+^IL-22^−^, [47.2–56.7] for IL-17A^+^IL-22^+^ and [43.8–56.6] for IL-17A^−^IL-22^+^, showing a comparable variability of polyfunctionality within all three T cell subsets according to Bartlett's test (*P* = 0.60).

### Application of the polyfunctionality index on Epstein-Barr Virus specific CD8^+^ T cells

**Figure 3:**
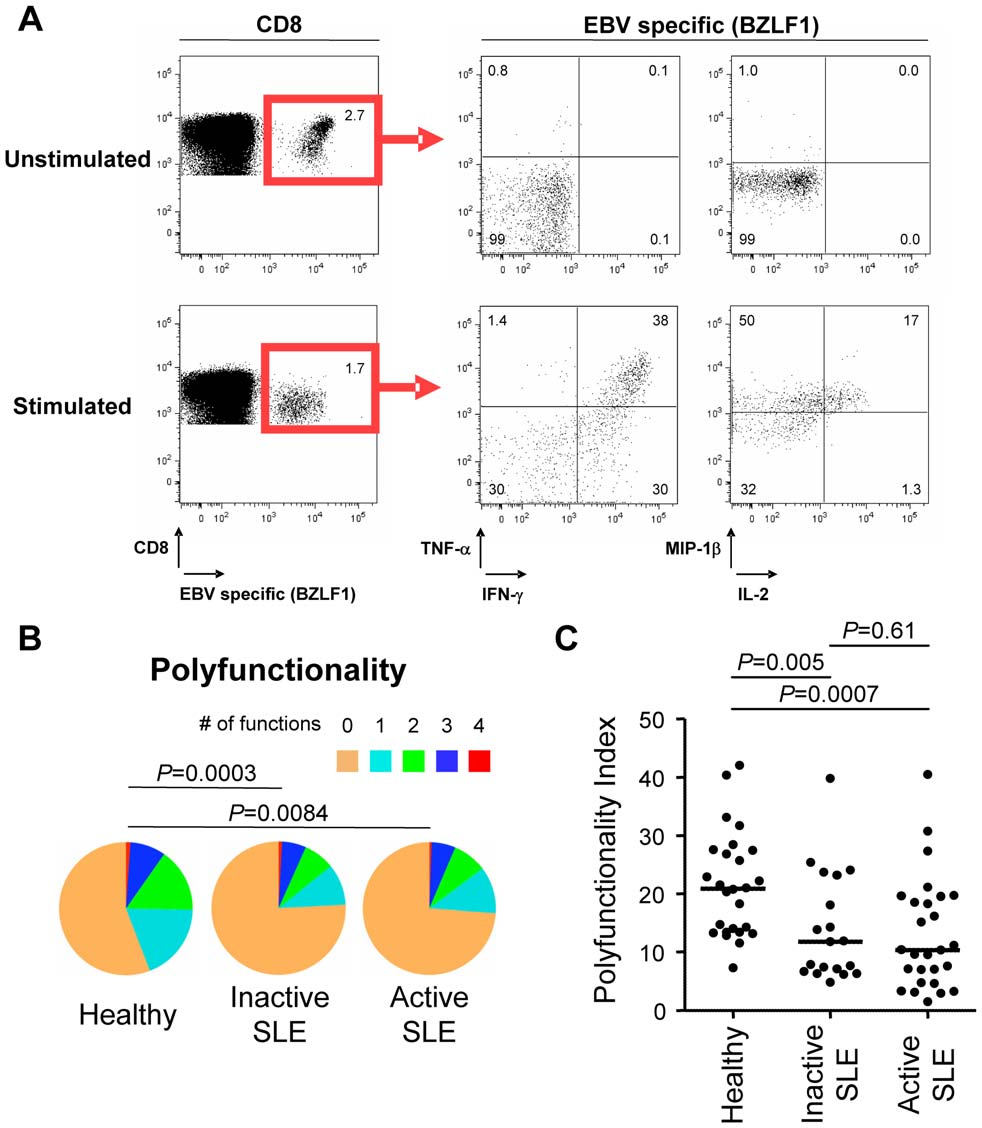
Characterization of EBV-specific CD8^+^ T cells from SLE patients and healthy controls. **A,** Representative cytofluorometric detection (left) and functional analysis (right) of CD8^+^ T cells specific for one of the lytic EBV antigens tested (BZLF1) pre (upper panel) and post (lower panel) peptide antigen stimulation of PBMCs from a healthy donor. Lytic EBV antigen-specific cells were detected with peptide/MHC tetramer and anti-CD8 antibody (red box) and simultaneously analyzed for intra-cellular IFN-γ, TNF-α, IL-2 and MIP-1β content. Cytokine/chemokine gates were positioned according to control stains of non-stimulated virus-specific T cells. **B,** EBV-specific T cells are strikingly less polyfunctional in 19 inactive (iSLE) and 27 active (aSLE) SLE patients compared to 26 controls (healthy). Pie representations of virus-specific CD8^+^ T cells represent the fraction of individual cells secreting none (0) or any (1, 2, 3 or 4) of the four cytokines IFN-γ, TNF-α, IL-2 and MIP-1β (color coded as indicated). E.g. the red pie slice indicates the proportion of cells producing four cytokines (IFN-γ, TNF-α, IL-2 and MIP-1β). **C,** Polyfunctionality index values of EBV-specific CD8^+^ T cells from inactive and active SLE patients as well as healthy controls. *P*-values monitoring differences between healthy donors and SLE patients are calculated using a non-parametric Mann-Whitney test and pie comparison statistics of the Spice software. Pie charts in B were adapted from our previous work. [11]

In a separate study we investigated whether raised EBV titres in Systemic Lupus Erythematosus (SLE) patients could be due to a functional impairment of EBV-specific CD8^+^ T cells. [11] We therefore measured the capacity of EBV-specific CD8^+^ T cells to secrete multiple effector-molecules (IFN-γ, TNF-α, IL-2 and MIP-1β) upon cognate antigen stimulation (Figure 3A). The polyfunctional profiles of EBV-specific CD8^+^ T cells from 19 inactive and 27 active SLE patients as well as 26 healthy controls were analyzed. The resulting pie chart representations showed a statistically significant different polyfunctionality profile of EBV-specific CD8^+^ T cells from inactive and active SLE patients compared to healthy controls (*P* = 0.0003 and *P* = 0.0084, respectively - Figure 3B). Statistical comparison of corresponding polyfunctionality index values pinpoints that these differences are a result of an impaired polyfunctionality of EBV-specific CD8^+^ T cells (*P* = 0.005 and *P* = 0.0007, respectively). In addition the polyfunctionality index evaluates the spread in polyfunctionality profiles for each group (Figure 3C).

### Correlative and cluster analysis of HIV-specific polyfunctional profiles

**Figure 4:**
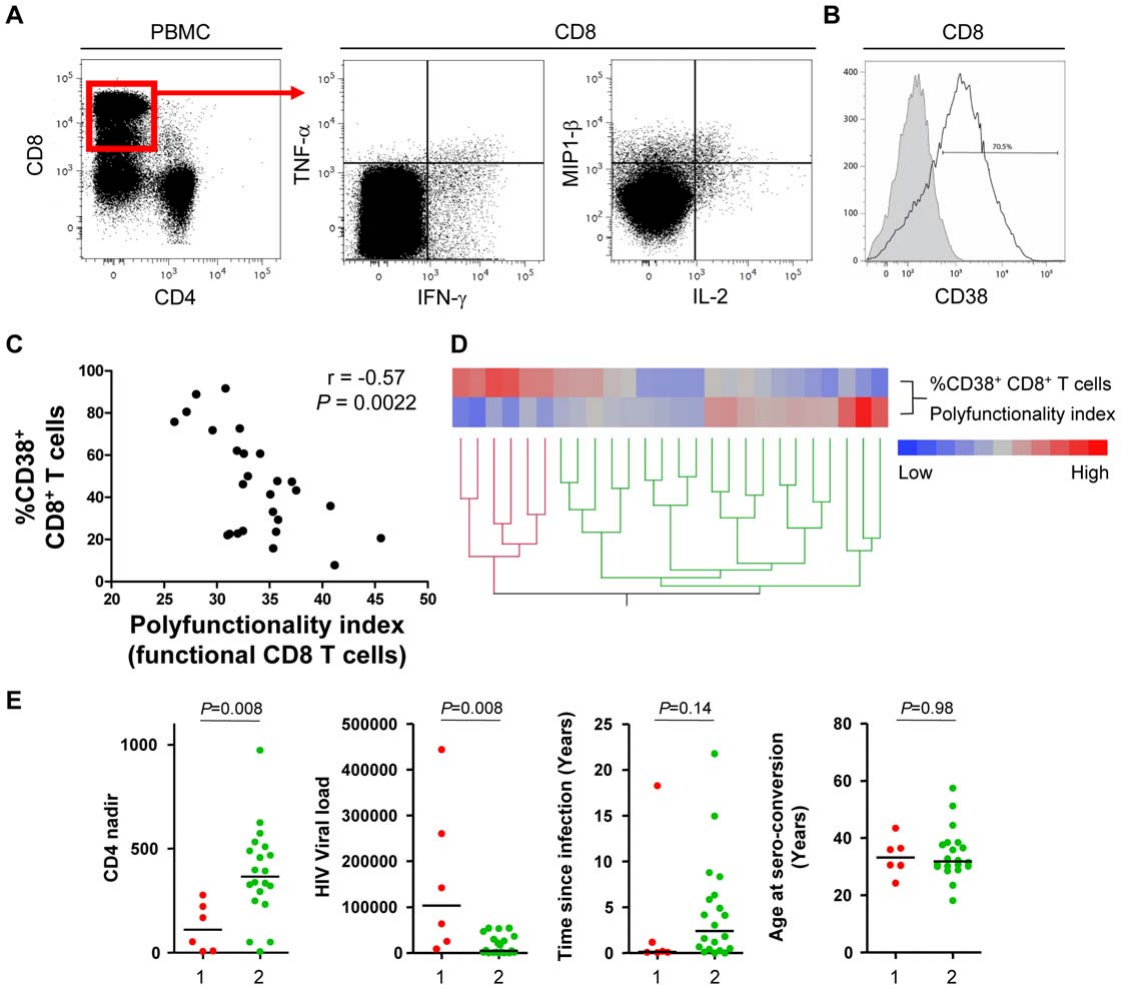
Associations of HIV-specific polyfunctionality and pathologically pertinent parameters. **A.** Representative cytofluorometric detection (left) and functional analysis (right) of CD8^+^ T cells post HIV-gag overlapping peptide stimulation of PBMCs from an untreated HIV-infected patient. CD8^+^ T cells (red box) were simultaneously analyzed for intra-cellular IFN-γ, TNF-α, IL-2 and MIP-1β content. Cytokine/chemokine gates were positioned according to control stains of non-stimulated CD8^+^ T cells. **B,** CD8^+^ T cell activation was monitored as CD38 surface expression (black line) versus isotype control (gray shade). **C.** Polyfunctionality index values correlate with CD8^+^ T cell activation for 26 HIV-infected donors. **D.** Hierarchical cluster analysis of CD8^+^ T cell activation and polyfunctionality generates a dendrogram that objectively separates HIV patients in 2 clusters. **E,** The two clusters are comprised of HIV patients with significantly different clinical status, defined by their CD4 nadir, HIV viral load, time since infection and age at seroconversion. *P*-values monitoring differences between clusters are calculated using a non-parametric Mann-Whitney test. Correlations were analyzed with a Spearman test.

To finally illustrate the importance of the polyfunctionality index in a clinical setting, we analyzed the polyfunctional CD8^+^ T cell response to HIV-gag antigen in a cohort of 26 untreated HIV-infected individuals (Figure 4A). The polyfunctionality index was calculated for HIV-gag responsive CD8^+^ T cells and analyzed for possible associations with biological and clinical parameters pertinent in HIV disease pathology. We previously demonstrated that T cell activation is associated with increased T cell expression of the negative functional regulator PD-1. [1] Moreover, PD-1 expression was associated with decreased T cell polyfunctionality. [11] In concordance with these results, we found that the polyfunctionality index was negatively correlated with T cell activation defined as the frequency of CD38^+^ CD8^+^ T cells (Figures 4B and 4C – r = −0.57, *P* = 0.0022). Subsequently, we investigated the importance of polyfunctional T cells on the association with CD8^+^ T cell activation. Indeed, the parameter *q* differentiates the impact of more or less polyfunctional T cells on the polyfunctionality index value. We therefore determined the *q* resulting in the best goodness of fit for the linear regression describing the association between the polyfunctionality index and CD8^+^ T cell activation (Figure 4C). A large *q-*value would indicate that highly polyfunctional T cells are of pivotal importance for the description of CD8^+^ T cell activation, whereas *q* close to 0 indicates that polyfunctionality has no or very little importance for the description of CD8^+^ T cell activation. Using a designated algorithm (see the Materials and Methods section) we found that *q* = 1.2 was optimal for associating polyfunctionality and CD8^+^ T cell activation. We furthermore illustrated this result by plotting the Pearson correlation coefficient (r) and the coefficient of determination (R^2^) as functions of *q* (Figure S2A). Importantly, *q* = 1.2 was also generating the correlation, with the highest degree of explanation (correlation coefficient closest to −1). The result was finally validated by a bootstrapped sensitivity analysis (see the Materials and Methods section), which found a very similar optimal *q*-value of 1.5 (Figure S2B). The result demonstrates that polyfunctionality is important for the description of CD8^+^ T cell activation, and that the impact increases approximately linearly with the number of featured functions.

We finally ordered patients according to a distance tree obtained through hierarchical cluster analysis to evaluate if CD8^+^ T cell activation and polyfunctionality could objectively distinguish patients according to their clinical status. The resulting dendrogram plot revealed that the largest distance change occurred between 1^st^ and 2^nd^ junctions, leading to the conclusion that patients can be objectively divided in two clusters (Figure 4D). The 1^st^ cluster includes patients with progressive HIV disease presenting high T cell activation and a low score of polyfunctionality combined with low CD4 nadir (the lowest CD4^+^ T cell count ever registered) and high HIV viral load. In contrast, the 2^nd^ cluster contains patients with stable HIV disease presenting low T cell activation and a high score of polyfunctionality combined with a high CD4 nadir (*P* = 0.008) and low viral load (*P* = 0.008) (Figure 4E).

## Discussion

The extensive advances in high throughput technologies over the last decades have generated heaps of data, leaving scientists with an acute need of accessible tools to analyse and present complex datasets. Multiparametric flow cytometry represent one such major advance in the field of cellular biology, which allows the acquisition of multiple phenotypic and functional parameters at the single-cell level. Most early studies employing this technology did not fully make use of the multiparametric character of the data. Instead, phenotypic and functional parameters of biologically relevant cell subsets were presented in a one-dimensional fashion. In recent years the development of fairly user-friendly bioinformatical software has paved the way for routine analysis of complex data. The software offer a comprehensible visual output of multiparametric datasets, which sufficiently retains the complexity of the data. [25] The impact of this type of analysis on the field of cellular biology cannot be overstated, however in the present study we postulate that a weighed one-dimensional index value represent an important supplement to *n*-dimensional complex representation of multiparametric data, such as polyfunctionality data.

We and others have previously measured polyfunctionality in one-dimension as the frequency of cells that perform all measured functions. [3], [26], [34] Although this method has been useful, it relies on often fairly low frequencies of cells performing all measured functions, and ignores information about all other functional T cell subsets. The very extensive reductionist approach applied by this method renders it less robust than methods taking into account all functional T cell subsets. Consequently, the polyfunctionality index was invented to reduce an n-dimensional polyfunctional profile to a one-dimensional value taking into account all cells performing 0, 1, 2 until n functions. The index comprises a weighed sum over *i* of the frequencies of T cells with *i* functions, where *i* ranges from 0 to *n*. The weights assigned to the individual functional T cell subsets increases with *i*. Therefore, the index assigns its higher values to the more polyfunctional profiles. Indeed the extremities represent completely non-functional cells (index value 0) and cells that all perform *n* functions (index value 100). The applied weights either increase with *n* in a linear or exponential manner. In the present study we have chosen the linear option, which is also the more conservative. Exponentially increasing weights should only be used when one have biological reasons to consider highly polyfunctional T cell subsets radically superior compared to less polyfunctional T cell subsets. Preferably, this should be validated with a mathematical approach. In the present study we found that the polyfunctionality index describes the activation of CD8^+^ T cells best for *q* = 1.2, which is in approximate concordance with the *q* = 1 applied throughout the present study. However, polyfunctionality may impact differently on other clinical or biological parameters, which should be addressed in future studies. Importantly, our study demonstrates that the impact of polyfunctionality may be objectively evaluated by combining the polyfunctionality index with automated iterative computations.

Any approach that reduces n dimensions to one represents a loss of information. Indeed, one polyfunctionality index value may represent multiple polyfunctionality profiles. Despite the loss of information the advantages of enumeration are many. In the present study we use three T cell functionality datasets to illustrate the analytical power of the polyfunctionality index. We previously demonstrated that IL-22-secreting CD4^+^ T cells have a different polyfunctionality profile than CD4^+^ T cells secreting IL-17A but not IL-22, when using IFN-γ, TNF-α and IL-2 secretion as functional markers. [15] In the present work we demonstrate that the polyfunctionality index assigns larger values to IL-22-secreting CD4^+^ T cells than IL-17A^+^IL-22^−^ CD4^+^ T cells, indicating that the former are the more polyfunctional. In another previous study we monitored the functional capacity of EBV-specific CD8^+^ T cells in SLE patients and healthy controls. [11] We demonstrated that EBV-specific CD8^+^ T cells from SLE patients have an aberrant polyfunctionality profile compared to healthy controls, when measuring IFN-γ-, TNF-α-, IL-2- and MIP-1β-secretion as functional parameters. In the present study we demonstrate that the observed difference is a result of polyfunctional impairment. Contrary to the pie chart representations, the polyfunctionality index makes it possible to evaluate the inherent variation of polyfunctionality across different donors and T cell subsets. In conclusion, the polyfunctionality index enumerates polyfunctionality and enables classical comparative statistics as well as the analysis of distribution statistics.

We finally demonstrate the feasibility of employing the polyfunctionality index in more sophisticated statistical analysis, such as correlative and hierarchical cluster analysis. Indeed, only a one-dimensional quantitative index value enables such analysis, contrary to qualitative representations, such as pie charts. The polyfunctionality index applied to the HIV-gag specific CD8^+^ T cell response in a cohort of untreated HIV-patients is significantly correlated with CD8^+^ T cell activation. We have previously demonstrated that negative functional regulation of T cells mediated by programmed death receptor 1 (PD-1) is closely correlated with T cell activation. [1] The negative correlation between T cell activation and polyfunctionality is therefore supporting our previous demonstration of activation induced negative regulation of T cells. Hierarchical cluster analysis of T cell activation and polyfunctionality furthermore demonstrate that the polyfunctionality index in combination with other pertinent parameters can delineate clusters of patients with distinct pathological profiles. Indeed, one unique cluster contains the majority of long-term non-progressors, defined here as patients several years post infection with high CD4 nadir and low HIV viral load. However, cluster analysis of polyfunctionality index and T cell activation alone does not separate long-term non-progressors from other non-progressive patients. Future studies on larger datasets should elucidate if the polyfunctionality index combined with other parameters could become predictive for HIV disease evolution, e.g. identification of long-term non-progressors. This underlines the important contributions a simple numerical score of polyfunctionality may have on diagnostics and prognostics of numerous disease pathologies and vaccine protocols.

The examples utilised in this study employed the special case of the “general polyfunctionality index”, where all measured functionalities are considered equally important. We therefore do not distinguish between parameters, e.g. considering IFN-γ secretion as more important than TNF-α secretion. Future studies should more thoroughly investigate if the ability of the “general polyfunctionality index” that allows discrimination between different functional parameters, could have biological and clinical impact. In conclusion, the polyfunctionality index efficiently changes polyfunctionality from a qualitative to a quantitative measure. We believe that future studies can readily include the polyfunctionality index as a potential end-point measurement, and include the parameter in more complex statistical analysis, such as correlation and hierarchical cluster analysis. Furthermore, mathematical evaluation of the relative impact of individual functions and their combinations on clinical and biological variables may be pivotal for our comprehension of polyfunctionality. In the present study we have successfully applied the polyfunctionality index to studies concerning cellular immunology. However, the potential scope of a polyfunctionality index reaches out to many other scientific areas, where multiple parameters are studied simultaneously at the single-cell level.

## Materials and Methods

### Polyfunctional analysis of human samples

The present work proposes a novel analytical tool for complex combinatorial datasets. It is essentially theoretical, but makes use of three experimental datasets to illustrate the power of the analysis. One experimental dataset was derived from a previously published study. [11] Of note, pie charts presented in Figure 3 derive from this study. The pro-inflammatory IL-17A- and IL-22-secreting CD4^+^ T cell responses of 25 healthy individuals were analyzed as previously described. [15] Similarly, HIV-specific responses of 26 untreated HIV-infected patients were analyzed by the same technical approach as previously described by us for CMV-specific responses. [38] The methodologies behind the datasets are therefore only briefly described here. Peripheral Blood Mononuclear Cells (PBMCs) were purified and stimulated with either 1 µg/ml PMA and 1 µg/ml Ionomycin (Sigma-Aldrich, France), EBV peptide antigen (5 µM) or HIV-gag p17 overlapping peptides (5 µM each) at 37°C. Cytokine secretion was blocked with 2.5 µg/ml monensin and 5 µg/ml brefeldin A (Sigma-Aldrich). After 16 h of stimulation, cells were incubated with directly conjugated anti-CD4-APC/Cy7 (BD Biosciences, San Jose, CA), anti-CD8-Alexa405 (Caltag, Burlingame, CA) and when appropriate PE-conjugated EBV peptide-MHC class I tetramer. Cells were permeabilized with Cytofix/Cytoperm™ (BD Biosciences) and stained with anti-cytokine antibodies for 20 minutes at room temperature. Finally, 10^6^ cell events were analyzed on a BD LSRII apparatus using FACSDiva (BD Biosciences) and FlowJo (TreeStar Inc) softwares. Polyfunctionality analysis was performed using Pestle Version 1.6.2 and Spice Version 4.2.3 (Mario Roederer, ImmunoTechnology Section, VRC/NIAID/NIH). [25]

### Ethics statement

Blood samples were obtained following acquisition of the study participants' written informed consent. The study protocol was reviewed and approved by the local ethic committee of Pitié-Salpêtrière Hospital, Paris.

### Statistical analysis

Differences between groups were tested using Mann-Whitney U-test (unpaired) and Wilcoxon signed rank test (paired). Correlations are analysed with a non-parametric Spearman test. All tests were 2-sided and a *P*-value<0.05 was considered statistically significant. Statistical analysis was performed using GraphPad Prism Ver. 4.03 (GraphPad Software Inc), Pestle Ver. 1.6.2 and Spice Ver. 4.2.3 (Mario Roederer, ImmunoTechnology Section, VRC/NIAID/NIH) softwares. [25]

### Goodness of fit analysis

We used the Generalized Reduced Gradient non-linear iterative computation included in the Frontline Systems Solver add-in for Microsoft Excel 2003 to find solutions for *q* resulting in the best goodness of fit for the linear association between polyfunctionality index values and CD8^+^ T cell activation. Graphical representation of the Pearson correlation coefficient (r) and the coefficient of determination (R^2^) as functions of *q* were obtained by tracking r and R^2^ for different *q*-values in Microsoft Excel 2003. To evaluate the robustness of the best goodness of fit calculation we combined a sensitivity analysis with multi bootstrapping using an add-in for Microsoft Excel 2003 available from www.wabash.edu/econometrics. Briefly, we calculated the R^2^ for the linear regression between CD8^+^ T cell activation and polyfunctionality index values for a panel of *q*-values ranging from 0.001 to 5.0 on 5000 bootstrapped datasets randomly generated by random draws with replacement (analysis repeated 5 times). The average r and R^2^ of these 5000 bootstrapped datasets were analyzed as a function of *q* (standard deviation was calculated for the 5 repeated analysis), and the *q* resulting in the largest R^2^ was identified.

### Hierarchical cluster analysis

Agglomerative hierarchical cluster analysis according to Ward's [43] was performed using the JMP7 software (SAS Software, NC, USA). The optimal number of clusters was identified according to the largest distance change between successive junctions of the dendrogram plot. Validity and reproducibility of the classification obtained with hierarchical cluster analysis was assessed using non-hierarchical k-means cluster analysis, in which the optimal number of clusters identified through hierarchical cluster analysis was pre-specified. Reproducibility of the classifications obtained with both hierarchical and non-hierarchical clustering was assessed by determination of the kappa value.

## Supporting Information

Figure S1 **Visualization of the polyfunctionality index for n = 2.** **A,** 2-Dimensional visualization of the polyfunctionality index for a system with maximal 2 functions (n = 2). Polyfunctionality index = ½F_1_+F_2_. **B,** Polyfunctionality index for a range of fictive T cells spanning the possible combinatorial frequencies (25% intervals) of T cells with zero, one and two functions respectively. The polyfunctionality index is calculated for *q* = 1 (black bars) and *q* = 2 (gray bars).
(PDF)

Figure S2 **Impact of** ***q*** **on the coefficients of correlation between polyfunctionality index and %CD38^+^ CD8^+^ T cells.** Plots of the Pearson correlation coefficient (r) and the coefficient of determination (R^2^) as functions of *q* based on **A.** the original dataset from the analysis of 26 HIV patients (cf. Figure 4) and **B.** the average of 5 bootstrap analysis performed for each *q* on the original dataset each comprising 5000 bootstrapped datasets. The error-bars represent the standard deviation of r- and R^2^-values. Dotted lines indicate the *q* value for which the coefficient of determination (R^2^) is maximal.
(PDF)

Appendix S1 **Description of the “General Polyfunctionality Index”.** Thorough description of the mathematical algorithm coined the “General Polyfunctionality Index” and its applications.
(PDF)
