# Supplementary figures and images for: Evaluating Cellular Polyfunctionality with a Novel Polyfunctionality Index
Source: PLoS One. 2012 Jul 30;7(7):e42403. doi: 10.1371/journal.pone.0042403 (PMC3408490; doi:10.1371/journal.pone.0042403)

Figure S1

A

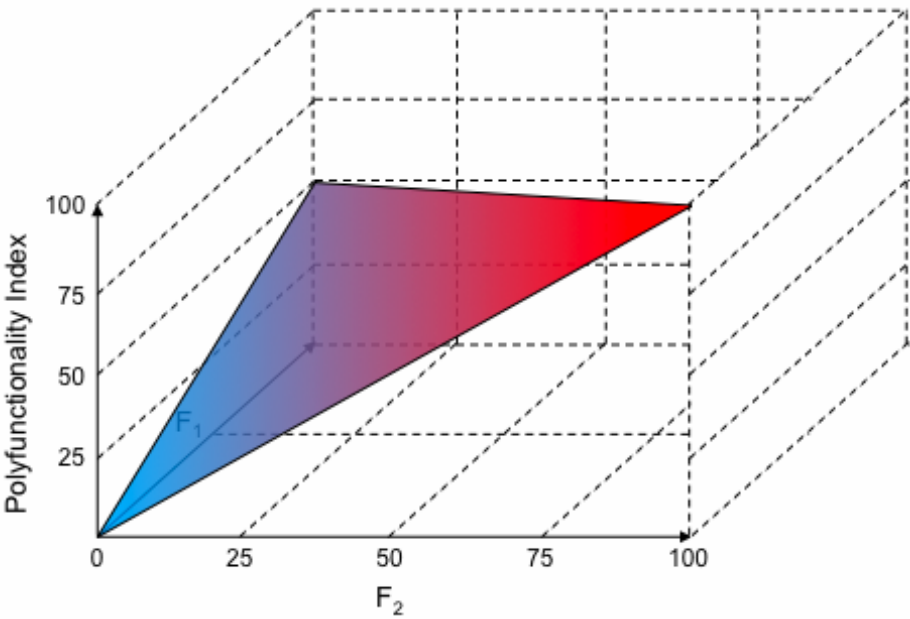

B

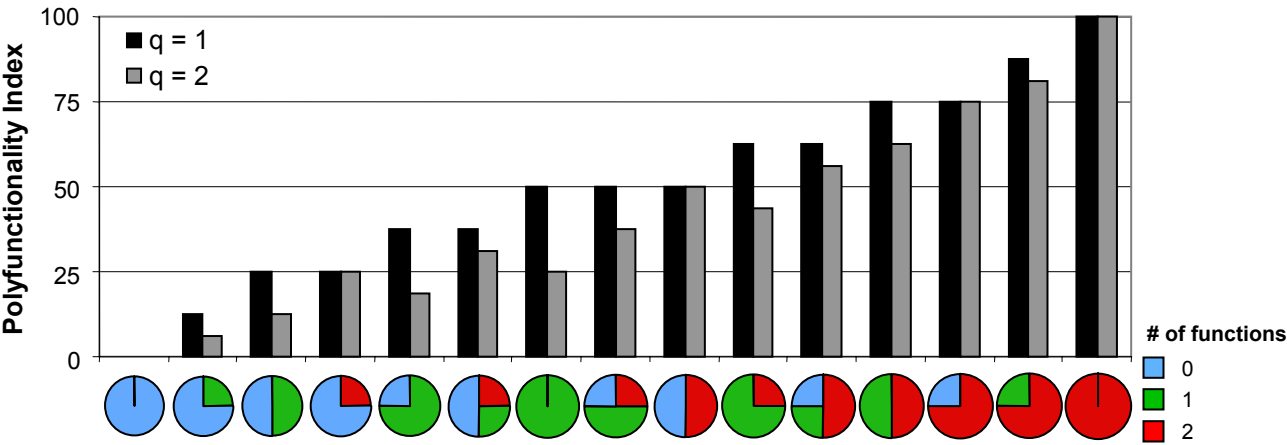

Supplement: Figure S1 — Visualization of the polyfunctionality index for n = 2. A, 2-Dimensional visualization of the polyfunctionality index for a system with maximal 2 functions (n = 2). Polyfunctionality index = ½F1+F2. B, Polyfunctionality index for a range of fictive T cells spanning the possible combinatorial frequencies (25% intervals) of T cells with zero, one and two functions respectively. The polyfunctionality index is calculated for q = 1 (black bars) and q = 2 (gray bars). (PDF) [file pone.0042403.s001.pdf]

Figure S2

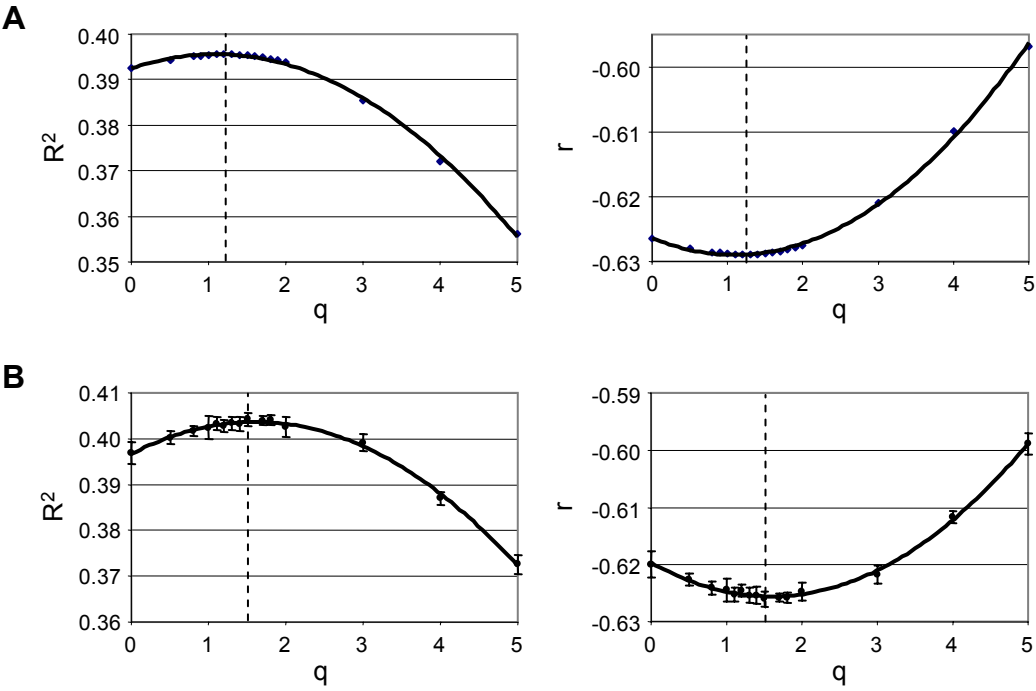

Supplement: Figure S2 — Impact of q on the coefficients of correlation between polyfunctionality index and %CD38+ CD8+ T cells. Plots of the Pearson correlation coefficient (r) and the coefficient of determination (R2) as functions of q based on A. the original dataset from the analysis of 26 HIV patients (cf. Figure 4) and B. the average of 5 bootstrap analysis performed for each q on the original dataset each comprising 5000 bootstrapped datasets. The error-bars represent the standard deviation of r- and R2-values. Dotted lines indicate the q value for which the coefficient of determination (R2) is maximal. (PDF) [file pone.0042403.s002.pdf]
