# Supplementary material for: Evaluating Cellular Polyfunctionality with a Novel Polyfunctionality Index
Source: PLoS One. 2012 Jul 30;7(7):e42403. doi: 10.1371/journal.pone.0042403 (PMC3408490; doi:10.1371/journal.pone.0042403)
Supplement: Appendix S1 — Description of the “General Polyfunctionality Index”. Thorough description of the mathematical algorithm coined the “General Polyfunctionality Index” and its applications. (PDF) [file pone.0042403.s003.pdf]

**Supporting Appendix S1: The "General Polyfunctionality Index"**

The combinatorial analytical approach employed for the analysis of multiparametric data, such as polyfunctionality of T cells, results by definition in an exhaustive dataset of $2^n$ dimensions. When this dataset is reduced to an n-dimensional polyfunctionality profile the information about which of the n functions contribute to the polyfunctionality are lost. E.g. when three functions are analysed simultaneously (n=3), the three combinations of functions constituting cells that perform 1 or 2 functions, respectively,(Figure 1A) are reduced by addition to obtain the frequencies of cells performing 1 and 2 functions (Figure 1B). In the further reduction to a 1-dimensional polyfunctionality index, we finally loose information about the individual frequencies of cells performing 1, 2 until n functions (Figure 1C). In return we obtain a simple degree of polyfunctionality.

Presently, there is no available mathematical approach to incorporate information about the contribution of the individual functions measured. Indeed, it is not possible to distinguish neither the polyfunctionality profile nor the index for donor #2 and #3 (Figures 1B-C). Here we evaluate a novel algorithm, coined the general polyfunctionality index, which allows that some functional measures are considered more important for polyfunctionality than others (4). We had the following requirements for the general polyfunctionality index. 1. The polyfunctionality index described in the main manuscript should be equal to the general polyfunctionality index when all functions have equivalent importance, 2. it should range from 0 to 100 and 3. it should be possible to assign different weight to all n functions.

$$\text{General Polyfunctionality index} = \frac{\sum_{x_1=0}^{1}\sum_{x_2=0}^{1}\cdots\cdots\sum_{x_n=0}^{1}\left(1+\varphi_{\begin{pmatrix}x_1\\x_2\\:\\x_n\end{pmatrix}}\right)\cdot F_{\begin{pmatrix}x_1\\x_2\\:\\x_n\end{pmatrix}}\cdot\left(\frac{\sum_{j=0}^{n}x_j}{n}\right)^q}{\left(1+\varphi_{\begin{pmatrix}1\\1\\:\\1\end{pmatrix}}\right)} \qquad (4)$$

$$x_j = 0 \text{ or } 1 \qquad (5)$$

$$\varphi_{\begin{pmatrix} x_1 \\ x_2 \\ \vdots \\ x_n \end{pmatrix}} = [\varphi_1 \quad \varphi_2 \quad \cdots \quad \varphi_n] \times \begin{bmatrix} x_1 \\ x_2 \\ \vdots \\ x_n \end{bmatrix} = \varphi_1 \cdot x_1 + \varphi_2 \cdot x_2 + \ldots + \varphi_n \cdot x_n \qquad (6)$$

$$\sum_{x_1=0}^{1} \sum_{x_2=0}^{1} \ldots \sum_{x_n=0}^{1} F_{\begin{pmatrix} x_1 \\ x_2 \\ \vdots \\ x_n \end{pmatrix}} = 100 \qquad (7)$$

$$F_{\begin{pmatrix} x_1 \\ x_2 \\ \vdots \\ x_n \end{pmatrix}} \geq 0 \text{ for all } x_j \qquad (8)$$

and $$\varphi_j \geq 0 \text{ for all } j \qquad (9)$$

In the above equations, $n > 0$ is the number of functions studied. $x_j$ indicates in a binary fashion (5) if the combinatorial T cell subset $\begin{pmatrix} x_1 \\ x_2 \\ \vdots \\ x_n \end{pmatrix}$ perform the j[th] function ($x_j = 1$) or not ($x_j = 0$). $F_{\begin{pmatrix} x_1 \\ x_2 \\ \vdots \\ x_n \end{pmatrix}}$ is the frequency (%) of cells performing the particular combination of functions $\begin{pmatrix} x_1 \\ x_2 \\ \vdots \\ x_n \end{pmatrix}$. $(1+\varphi_{\begin{pmatrix} x_1 \\ x_2 \\ \vdots \\ x_n \end{pmatrix}})$ is the differential weight assigned to a T cell subset performing the particular combination of functions $\begin{pmatrix} x_1 \\ x_2 \\ \vdots \\ x_n \end{pmatrix}$ (6). This particular T cell subset performs $\sum_{j=0}^{n} x_j$ functions, and is additionally assigned the weight also applied for the general polyfunctionality index, $\left( \frac{\sum_{j=0}^{n} x_j}{n} \right)^q$, for which $q \geq 0$ is the parameter that modulates the

differential weight assignment of each $F_{\begin{pmatrix} x_1 \\ x_2 \\ \vdots \\ x_n \end{pmatrix}}$ in a linear ($q$=1) or exponential ($q \neq 1$) manner. The algorithm requires that the sum of all $F_{\begin{pmatrix} x_1 \\ x_2 \\ \vdots \\ x_n \end{pmatrix}}$ equals 100 (7) and that all $F_{\begin{pmatrix} x_1 \\ x_2 \\ \vdots \\ x_n \end{pmatrix}}$ are absolute values (8). To restrain the general polyfunctionality index to values between 0 and 100, we impose that all differential weights ($\varphi_j$) are absolute values (9) and normalize by dividing with the largest theoretical index value obtained by cells that perform all n functions, $(1 + \varphi_{\begin{pmatrix} 1 \\ 1 \\ \vdots \\ 1 \end{pmatrix}})$.

To exemplify the general polyfunctionality index we make use of the theoretical example (n=3 and $q$=1) of donors #1, #2 and #3 (Figure 1). In this case we will increase the importance of IFN-γ-secretion ($\varphi_{IFN\text{-}\gamma} = 0.5$), compared to TNF-α- and IL-2-secretion ($\varphi_{TNF\text{-}\alpha} = \varphi_{IL\text{-}2} = 0$).

$$\varphi_{\begin{pmatrix} x_{IFN-\gamma} \\ x_{TNF-\alpha} \\ x_{IL-2} \end{pmatrix}} = \begin{bmatrix} 0.5 & 0 & 0 \end{bmatrix} \times \begin{bmatrix} x_{IFN-\gamma} \\ x_{TNF-\alpha} \\ x_{IL-2} \end{bmatrix} = 0.5 \cdot x_{IFN-\gamma}$$

$$\text{General polyfunctionality index} = \sum_{x_{IFN-\gamma}=0}^{1} \sum_{x_{TNF-\alpha}=0}^{1} \sum_{x_{IL-2}=0}^{1} (1+\varphi_{\begin{pmatrix} x_{IFN-\gamma} \\ x_{TNF-\alpha} \\ x_{IL-2} \end{pmatrix}}) \cdot F_{\begin{pmatrix} x_{IFN-\gamma} \\ x_{TNF-\alpha} \\ x_{IL-2} \end{pmatrix}} \cdot \frac{\sum_{j=0}^{3} x_j}{3}$$

Donor #1, #2 and #3 hereby obtain general polyfunctionality index values of 50, 50 and 44.4, respectively (Figure 1D). This illustrates that adding weight to IFN-γ-secretion allows us to distinguish between donor #2 and #3, favoring donor #2 for its higher content of IFN-γ-secreting cells. The general polyfunctionality index of donor #1 and donor #2 remain identical, because functional T cells from these two donors all secrete IFN-γ.
